# Implementation and Evaluation of Four Interoperable Open Standards for the Internet of Things

**DOI:** 10.3390/s150924343

**Published:** 2015-09-22

**Authors:** Mohammad Ali Jazayeri, Steve H. L. Liang, Chih-Yuan Huang

**Affiliations:** 1 Department of Geomatics Engineering, University of Calgary, 2500 University Drive NW, Calgary, AB T2N 1N4, Canada; E-Mails: sma.jazayeri@gmail.com (M.A.J.); steve.liang@ucalgary.ca (S.H.L.L.); 2 Center for Space and Remote Sensing Research, National Central University, No. 300, Jhongda Rd., Jhongli District, Taoyuan City 320, Taiwan

**Keywords:** Internet of Things, interoperability, sensor, IoT device, OGC SWE, Sensor Observation Service, PUCK, CoAP, SensorThings

## Abstract

Recently, researchers are focusing on a new use of the Internet called the Internet of Things (IoT), in which enabled electronic devices can be remotely accessed over the Internet. As the realization of IoT concept is still in its early stages, manufacturers of Internet-connected devices and IoT web service providers are defining their proprietary protocols based on their targeted applications. Consequently, IoT becomes heterogeneous in terms of hardware capabilities and communication protocols. Addressing these heterogeneities by following open standards is a necessary step to communicate with various IoT devices. In this research, we assess the feasibility of applying existing open standards on resource-constrained IoT devices. The standard protocols developed in this research are OGC PUCK over Bluetooth, TinySOS, SOS over CoAP, and OGC SensorThings API. We believe that by hosting open standard protocols on IoT devices, not only do the devices become self-describable, self-contained, and interoperable, but innovative applications can also be easily developed with standardized interfaces. In addition, we use memory consumption, request message size, response message size, and response latency to benchmark the efficiency of the implemented protocols. In all, this research presents and evaluates standard-based solutions to better understand the feasibility of applying existing standards to the IoT vision.

## 1. Introduction

### 1.1. Background

Internet-connected services are growing rapidly. A great number of people use the Internet for surfing the web, accessing multimedia, sending and receiving emails, playing games, shopping, social networking and many other daily tasks. Consequently, the Internet can intuitively be a good candidate for involving citizens in sensing systems. Therefore, the concept of the Internet of Things (IoT) emerged as a networking infrastructure to interconnect electronic objects with the Internet as a medium.

**Figure 1:**
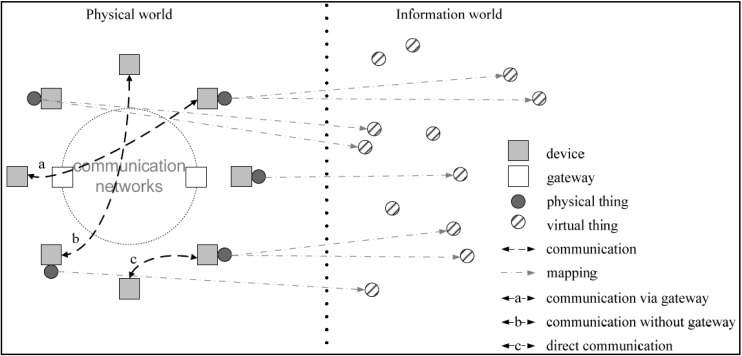
Technical overview of the IoT (Source: Recommendation ITU-T Y.2060—Overview of the Internet of Things [1]).

In this research, we follow the IoT definition provided by International Telecommunication Union (ITU) [1]: “Internet of Things is a global infrastructure for the information society, enabling advanced services by interconnecting (physical and virtual) things based on existing and evolving interoperable information and communication technologies”. Figure 1 depicts this concept by mapping the physical world to the digital world across communication networks. With the ubiquitous nature of the Internet, people will be able to monitor and control Internet-connected objects from anywhere through the IoT infrastructure. Although there has not been a clear IoT infrastructure developed, the strong potential of the IoT has led to many proposals for IoT applications.

In reference to the ITU definition, a *Thing* is described as a uniquely identifiable instance of the physical world or the information world, which can be integrated into communication networks [1]. For this research, we focus on physical devices with the mandatory capabilities of communication and the optional features of sensing, actuation, data capture, data storage and data processing. Bormann *et al.* [2] from IETF (Internet Engineering Task Force) analyzed and categorized IoT objects into three categories with respect to their communication capabilities: *class-0* devices (*i.e.*, too small to securely run on the Internet), *class-1* devices (*i.e.*, devices with about 10 KB of RAM and 100 KB of code space), and *class-2* devices (*i.e.*, devices with about 50 KB of RAM and 250 KB of code space). Bormann *et al.* [2] argue that the class-0 devices require extra help to communicate with other devices; the class-1 devices cannot easily communicate with other devices or applications through traditional XML-data representations and protocols (e.g., HTTP and Transport Layer Security (TLS)); and the class-2 devices are able to communicate with the traditional transfer protocols and data encodings. Based on these definitions, we believe that the relatively inexpensive class-1 devices would be a reasonable choice for applications in the IoT infrastructure. Thus, this research focuses on the class-1 devices. This way, we can estimate the lowest boundary of resources required for IoT devices, as well as ensure that our proposed solutions are feasible and economically effective for real-world applications.

While the IoT concept has attracted increasing attention from various domains, there remain some critical issues to be addressed. In this research, we identify and emphasize two major issues of the IoT. Firstly, for IoT devices to easily *plug-and-play* [3], each IoT device needs to be *self-describable* and *self-contained* in order to communicate with other objects or services. That is, a Thing should be able to describe and advertise itself and its capabilities, which in general is the metadata of the Thing. Secondly, while current Internet-connected IoT devices are mainly developed for specific applications, their communication protocols and their data encodings are usually proprietary and different from each other. For example, Air Quality Egg and Ninja Block are sensor systems designed to collect high resolution observations of environmental dynamics; however, their hardware design and their data communication are completely different. This lack of *heterogeneity* obstructs the communication and cooperation between the IoT devices with different applications. Whilst there are other important issues in the IoT infrastructure, such as limited power supply, privacy and security concerns, they will be discussed in the future.

With regards to the first issue mentioned above, for IoT devices to be self-describable and self-contained, one feasible solution is to provide web services on the IoT devices. The web services provided can advertise the capabilities and information of the devices in the network. For the second issue, we need to consider the communication interoperability between IoT devices, web services, and applications. Based on the IEEE definition [3], *syntactic interoperability* means the ability of interoperation and information exchange in a system; which is that devices should be able to interactively communicate with a common protocol and data format. Besides the syntactic interoperability, devices should exhibit *semantic interoperability* as well. If devices are semantically interoperable, they are able to interpret the exchanged data and understand each other. Although interoperability has a broader scope, we will focus only on the syntactic interoperability in this research.

According to Rodriguez *et al.* [4], Sensor Web and Wireless Sensor Networks (WSNs) play an important role in the IoT infrastructure. One possible solution, for providing global interoperability for all IoT devices is to follow Sensor Web open standards. One of the pioneers in the Sensor Web standardization is the *Open Geospatial Consortium* (*OGC*). OGC has been supporting geospatial interoperability since 1994. One of the OGC standards, the Sensor Web Enablement (SWE), is a suite of standards designed to achieve sensor network interoperability. SWE standards include *Observations & Measurements* (*O&M*) [5], *Sensor Model Language* (*SensorML*) [6], *Sensor Observation Service* (*SOS*) [7], *Sensor Planning Service* (*SPS*) [8], *PUCK* protocol [9], and SensorThings, which is specifically designed for IoT and is currently in the finalization stage.

The O&M defines standard models and XML schema for observations and measurements collected by sensors. The SensorML specification includes standard models and XML schema for representing the metadata of sensor systems and processes. SOS presents a standard web service interface for requesting, filtering, and retrieving observations and sensor system information. A SOS service is an intermediary between a client and sensor observation repositories. The SPS specification provides a standard web service interface for users to make observations with sensors. The PUCK standard introduces a low-level protocol to retrieve SensorML documents, sensor driver code, and other information from sensors. SensorThings defines a standard way to interconnect IoT devices, data servers, and applications over the Web through a REST-like API.

Intuitively, one possible solution to achieve the interoperable IoT is to apply the OGC open standards on the IoT devices. However, as most of the SWE standards are designed for large-scale sensor arrays rather than for resource-constrained IoT devices, this research proposes some mechanisms to host SWE services on IoT devices. As such, one of our contributions is implementing the SWE standards on IoT devices and performing feasibility analysis to evaluate whether these standards are suitable for the IoT. Apart from the OGC standards, there are other standards that can be developed on IoT devices such as, *Constrained Application Protocol* (*CoAP*) [10]. This protocol efficiently employs the basic features of HTTP to the constrained networks and devices. In addition, this research also implements and evaluates the use of CoAP on IoT devices.

### 1.2. Objectives

The main objective of this research is to address the IoT interoperability issue. To achieve this goal, we implement four standard-based solutions on class-1 IoT devices including (1) PUCK over Bluetooth; (2) TinySOS; (3) SOS over CoAP; and (4) OGC SensorThings. These four solutions are respectively explained in Section 2, Section 3, Section 4 and Section 5. In order to provide enough detailed information for each solution, each section contains an introduction, literature review and proposed system architecture. Section 6 then evaluates and compares the performance of the four implemented solutions. Finally, conclusions and information on future work are given in Section 7.

To summarize, the major contribution of this research is to explore the possible approaches to achieving interoperability between class-1 IoT devices. At the end, we complete our contribution by evaluating the performance of the applied standards in terms of memory occupation (ROM and RAM), request length, response size and response latency. We expect the direction addressed in this research to be a motive to establish a better infrastructure for the future of the IoT.

## 2. PUCK over Bluetooth

### 2.1. Introduction

Within the scope of the OGC standards, we first choose PUCK, which is a simple command protocol. The PUCK contains a set of standard commands to access the device memory, read the device metadata, and write data on the memory. The prime purpose of the OGC PUCK is to provide interoperability for devices connected through serial cables or Ethernet. In order to enable sensors to be accessible via wireless connections, we analyze possible radio communication technologies. The choice of the radio is highly important since it influences either energy consumption or software design. Compared to Zigbee and RF transceiver alternatives which are applied in WSNs or Sensor Webs, Bluetooth is more popular because it is widely supported by many daily devices (e.g., cell phones and notebooks). In addition, Bluetooth is more energy-efficient in comparison to Wi-Fi. As a result, we integrate the Bluetooth protocols to the PUCK standard in order to raise the interoperability between various types of sensors and actuators, namely IoT devices.

**Figure 2:**
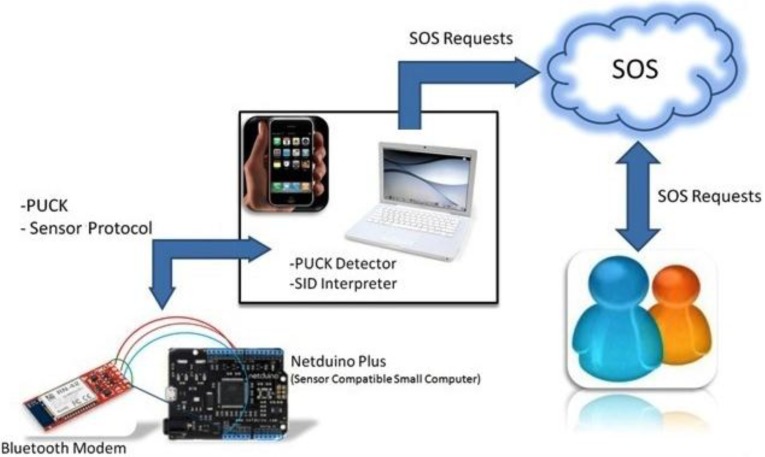
The overall workflow for accessing the sensor measurements.

PUCK standard is efficiently designed to be applied on devices supporting different protocols. It includes two modes: *PUCK mode* for processing the PUCK commands, and *instrument mode* for handling instrument-specific operations. Since the PUCK itself has no support for retrieving and publishing sensor measurements on the Internet, we use additional software components and the OGC SOS to provide users a standard access to the sensor observations. The workflow is shown in Figure 2.

According to Figure 2, a client first runs the *PUCK Detector* application to discover PUCK-enabled devices. The PUCK Detector then sends out a PUCK command to devices which are in the PUCK mode. After successful discovery, a connection is established via Bluetooth and the client can send other PUCK commands to the device. Since PUCK provides access to data saved in the PUCK memory, it does not support communications in the device protocol. In order to handle such communications, we apply another OGC standard named *Sensor Interface Descriptor* (*SID*) [11]. Firstly, a SID file is stored on the device which includes the description of the device protocol. Then, the client uses the SID Interpreter [11] to retrieve sensor readings in the instrument mode, and finally the data is uploaded to an online SOS.

To sum up, the first contribution of this section is that we initially enable sensors to be accessible through Bluetooth technology. Then, we integrate Bluetooth protocol and PUCK as an open standard wireless protocol to increase the interoperability of IoT devices.

### 2.2. Related Work

Bluetooth has already been utilized in Sensor Web [12] to allow sensors to upload their readings to a data repository. Leopard *et al.* [13] achieved this by introducing a tiny Bluetooth stack that allows TinyOS [14] applications to be executed on Bluetooth-enabled sensor nodes. Whilst Leopard *et al.* [13] focused on efficient network processing and system architecture design, their research did not consider the interoperability issues between various sensors.

Since the Bluetooth radio range is over a couple of meters [15], the system developed by Leopard *et al.* [13] does not provide worldwide access to the sensor measurements. To overcome this problem, Ferrari *et al.* [16] proposed a new architecture for the sensor networks to integrate the Bluetooth-enabled sensors with Internet-connected computers. As a result, these Bluetooth-enabled sensors become connected to the Internet. Although this implementation successfully demonstrated the possibility of combining Bluetooth sensor nodes to the web interfaces, the communication protocol between sensors and computers was proprietary and did not consider interoperability issues.

As far as we know, there is no standard protocol based on Bluetooth that enables IoT devices to be connected in an interoperable manner. We believe that the integration of Bluetooth and the OGC standards for IoT devices can be a pioneering development in this field.

### 2.3. Architecture

We briefly introduce the OGC PUCK protocol here. Next, we explain the sensor protocol for retrieving sensor observations from the device. Finally, we present the high-level architecture of our proposed system.

#### 2.3.1. Sensor Protocol

**Figure 3:**
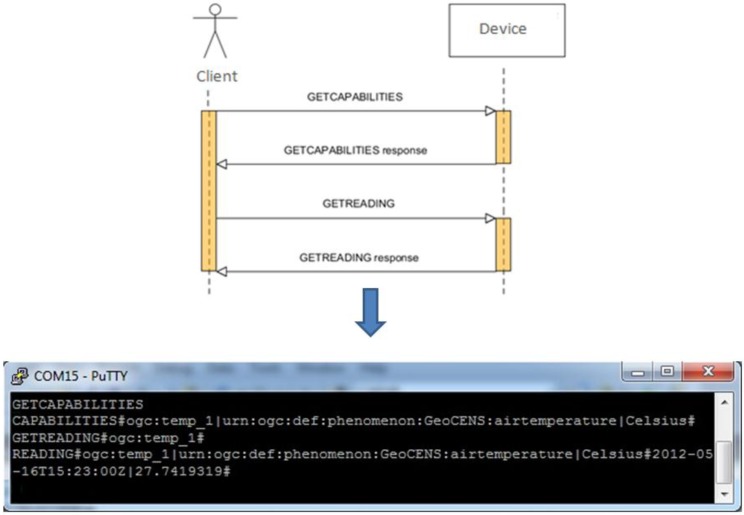
Procedures of the sensor protocol.

The purpose of the sensor protocol is to allow users to simply query sensor capabilities, observations, and presentations of observed features in the instrument mode. Figure 3 depicts the procedures of the sensor protocol.

We designed the sensor protocol based on the concept of OGC SOS [7] to serve the demonstration purpose. Most of the terms used in this part follow the terminology in the OGC SOS. Because of the limited resources for IoT instruments, the command and response formats should be considered as simply as possible. Therefore, unlike the SOS applying XML as the format, this protocol simply defines “separators” (e.g., {#, :, |}) to format requests and responses (Figure 3). Similarly with the OGC SOS, we define the *GETCAPABILITIES* operation in order to show the capabilities of the device. The response includes the unique IDs of the sensors attached to the device, the phenomena IDs which are measured by the sensors, and the unit of measurements. Then, the other operation, *GETREADING*, can be sent to retrieve sensor readings.

#### 2.3.2. System Architecture

**Figure 4:**
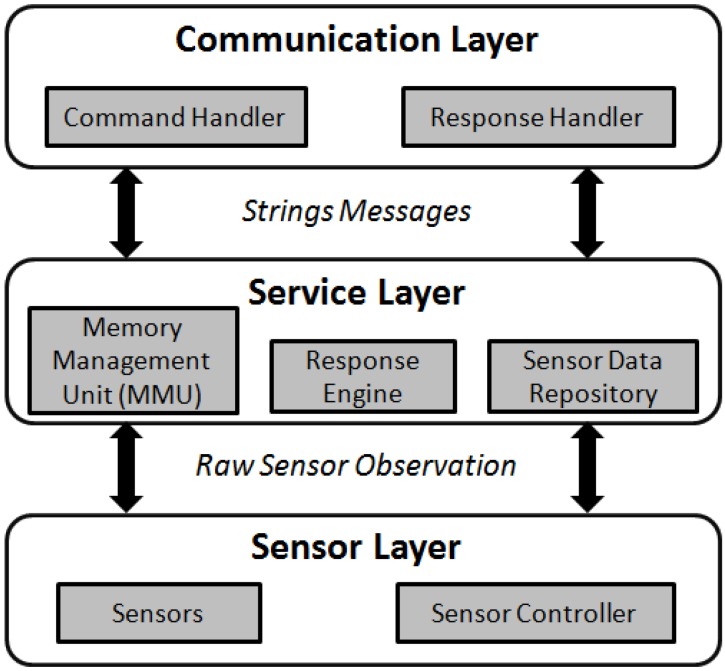
The system architecture supporting the PUCK protocol.

As shown in Figure 4, the architecture we proposed for the device follows a layered structure which has three major layers: *Communication Layer*, *Service Layer*, and *Sensor Layer*.

- *Communication Layer*: This layer includes the Bluetooth hardware and its protocol. When a request is received, the layer forwards the request string to the service layer for processing. After the service layer finishes processing the request, a response string is returned to the communication layer to be sent back to the client.
- *Service Layer*: The service layer handles business logic of the system. This layer itself consists of three modules: *sensor data repository*, *response engine*, and *memory management unit* (*MMU*). Sensor data repository archives historical records of sensor observations. In order to parse the commands and compose the response messages on low-memory IoT devices, we propose a response engine. This unit is equipped with a *buffering* mechanism to handle large requests and responses. In this way, the maximum memory consumption at any time for reading and writing a document is equal to the buffer size. For our implementation, the buffer size is considered 1 KB which is more than enough for the commands of PUCK and sensor protocol. When the request is processed by the response engine, the response comes from the MMU (if the command relates to PUCK memory), or from the sensor data repository (if the request contributes to the sensor protocol). By following this technique, we can successively parse and compose large commands (~100 KB) on class-1 IoT devices.
- *Sensor Layer*: The sensor layer consists of physical sensors and a sensor controller. The sensor controller tasks sensors to collect sensor observations. Next, it sends the collected sensor observations to the sensor data repository of the service layer.

## 3. TinySOS

### 3.1. Introduction

This section addresses the issues from the *decentralized* and *heterogeneous* nature of IoT objects and sensors. The main idea is inspired by two papers published by Priyantha *et al.* [17] and Bormann *et al.* [2]. Priyantha *et al.* [17] proposed a *tiny web service* for sensors and an application-level interface which provide three advantages. Firstly, each sensor becomes self-describable and self-contained by providing web interfaces for applications to retrieve sensor’s capabilities. It means the device does not depend on other network nodes (e.g., hosts) to publish its data or to receive requests. Secondly, some form of privacy is preserved for device owners through the direct connection to their devices. In addition, sensor deployment and maintenance are easier with the interfaces for updating a sensor’s metadata. However, in order to achieve the interoperability between sensors and applications, one solution is to using standard-based web service interfaces and widely-used data encodings in information communication.

To the best of our knowledge, there is no existing work that evaluates the feasibility of constructing a SWE web service directly on an object with limited resources. This evaluation is important because if SWE web services can be hosted on IoT devices, the IoT devices will not only be self-describable and self-contained, but they will also directly inherit the comprehensive SWE conceptual model. In that case, the IoT objects can interoperate with each other as well as the existing OGC SWE applications. Moreover, some form of privacy might be preserved by removing the gateways in the path between the applications and devices.

Among the SWE specifications, we choose the Sensor Observation Service (SOS), which defines a web service interface for accessing sensor observations and metadata [7], to be implemented on a class-1 IoT device. Our implementation of the SOS is termed *TinySOS* [18] that supports a lightweight profile of the OGC SOS suitable for resource-constrained IoT devices. Since SOS is mainly based on XML documents which could be too large to be managed in the limited memory of the class-1 devices, we propose the XML processor unit (XPU) in order to process XML documents buffer by buffer.

### 3.2. Related Work

There have been some existing IoT projects applying proprietary protocols, such as Microsoft’s HomeOS [19], Xively (previously known as Cosm and before that Pachube), MicroStrain’s SensorCloud, and Wovyn. Many of them provide a web portal for users to manipulate the data collected by their sensors. We refer to these web portals as *the IoT portals*. Most of the IoT portals allow users to visualize the time-series data collected by sensors or publish the data with their own *Application Programming Interfaces* (*APIs*). However, in this case, IoT objects, that support only one type of proprietary APIs, form a “silo”, and cannot interoperate with objects in other silos. Consequently, the development of various IoT silos obstructs the development of the IoT. Therefore, in order to break down these silos and achieve the vision of an open IoT environment, following open standard protocols is necessary.

There have been some initiatives for integrating SWE and IoT. For example, presentations and talks such as “SWE and IoT” [20], “Sensor Web Standards and the IoT” [21], “Bringing IoT to the mass market—what should a standard do?” [22], and “Collaborative development of open standards for expanding GeoWeb to the Internet of Things” [23] were given in workshops and OGC Technical Committee meetings to discuss the possibility of applying SWE standards to the IoT. Moreover, Broring *et al.* [24] implemented *SenseBox*, which utilizes the O&M standard in their web service API. However, the web service on their SenseBox does not follow SWE standards. Furthermore, Resch *et al.* [25] also implemented SWE standards (including SOS) on an embedded sensing device. However, their sensor hardware has 512 MB RAM and 32 MB flash memory, which is even more powerful than the class-2 device mentioned in Bormann *et al.* [2]. We argue through that it is still necessary to evaluate the feasibility of implementing SWE standards on a relatively inexpensive class-1 device.

### 3.3. Architecture

In this section, we introduce a lightweight profile of the SOS—TinySOS. Next, we present the high-level system architecture of TinySOS for class-1 IoT devices.

#### 3.3.1. TinySOS

In order to host web services on class-1 devices, the web service needs to be sufficiently lightweight. Therefore, for this implementation, we only selected the mandatory operations of the SOS (*i.e.*, the core operations) for the TinySOS. There are three mandatory operations in the OGC SOS named *GetCapabilities, DescribeSensor*, and *GetObservation*.

The *GetCapabilities* operation provides access to metadata and detailed information about the available capabilities of the service. The *GetCapabilities* request can be sent either by HTTP GET or POST request type to retrieve the service metadata as an XML file (*i.e.*, the Capabilities document). The XML file contains metadata about this service, such as unique sensor identifiers, logical groupings of sensor observations (*i.e.*, the ObservationOfferings in the SWE terminology), and the URIs of physical phenomena (*i.e.*, the ObservedProperties) that sensors are measuring. Users can use the information in the Capabilities document to retrieve the sensor metadata and the observations with the other two core operations.

The *DescribeSensor* operation allows users to retrieve sensor metadata with a unique sensor identifier specified in the Capabilities document. If the *DescribeSensor* request is valid (*i.e.*, the service has sensors that match the unique identifier), the SOS returns the sensor metadata in the SensorML format.

The *GetObservation* operation provides access to the observations made by the sensors. Users can use the ObservationOffering and ObservedProperty in the *GetObservation* request as criteria in querying sensor observations. According to the criteria specified in the request, the SOS returns the sensor observations in the O&M format.

#### 3.3.2. System Architecture

**Figure 5:**
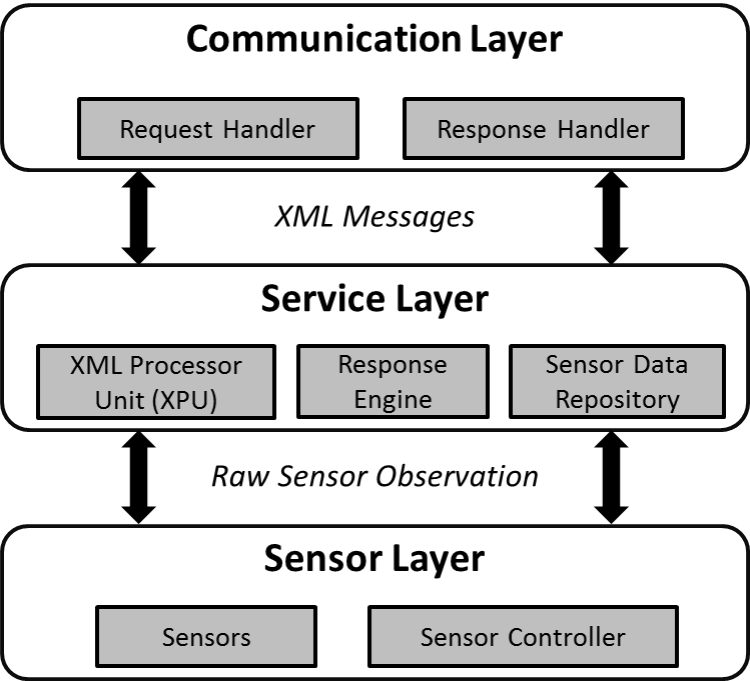
The system architecture supporting the TinySOS protocol [18].

As we saw from the previous sub-section, to support the three core operations of the SOS, an IoT device needs the functionalities to validate the HTTP request type (*i.e.*, GET, POST) and content type (*i.e.*, text/xml), in order to parse the XML request, and to create the XML response. To achieve these functionalities, we present the proposed system architecture of the TinySOS. There are three major layers in the TinySOS service (Figure 5), including *Communication Layer*, *Service Layer*, and *Sensor Layer*.

- *Communication Layer:* The communication layer is responsible for managing HTTP requests and responses, including the network related protocols and hardware (e.g., Network Interface Card). When a request is received by a TinySOS service, the communication layer forwards the XML request to the service layer for further processing. After the service layer finishes the task, an XML response is returned to the communication layer, and then sent back to the client.
- *Service Layer:* This layer consists of three modules: *XML processor unit* (*XPU*), *response engine*, and *sensor data repository*. As the XML documents are significantly too large to be stored in the memory of class-1 devices, the TinySOS service needs a new way to parse XML documents. Instead of the traditional XML parsers that load the whole XML document into memory, we propose the use of the XML processor unit (XPU) which reads and parses XML documents buffer by buffer. The XPU not only extracts the request criteria parameters, but also composes the *GetObservation* responses. The request criteria extracted by the XPU is forwarded to the response engine. If it is a *GetCapabilities* request or a *DescribeSensor* request, the response engine retrieves a predefined XML file (e.g., the Capabilities document and the SensorMLs) from the permanent memory, and forwards it to the communication layer. If the request is a *GetObservation* operation, the response engine uses the XPU to compose the *GetObservation* response according to the criteria, and forwards the response to the communication layer. In addition, as an SOS should have the ability to return the historical observations, the TinySOS stores sensor measurements in a sensor data repository. Depending on the device, the sensor data repository could be located in either the main memory (RAM) or the permanent memory (e.g., micro SD card).
- *Sensor Layer:* The sensor layer consists of the physical sensors and the sensor controllers. The sensor controllers work closely with sensors. For example, a sensor controller can task sensors to collect sensor observations and send the collected sensor observations to the sensor data repository in the service layer. The sensor controller would play an important role in supporting SPS on IoT objects.

## 4. SOS over CoAP

### 4.1. Introduction

IoT devices are usually limited in power, network, memory and processing capabilities [2]. The aforementioned standard protocols, PUCK and SOS, have not typically been designed with power and network efficiency in mind. The naive solution is forcing the battery-powered device to keep its radio off as much as possible. Another solution is to minimize the network load which not only significantly saves bandwidth, but also the radio transceiver can fulfill its task faster resulting in more sleeping [26].

To achieve this, we selected the IETF protocol designed for constrained nodes and networks (e.g., WSNs), which is named *Constrained Application Protocol* (*CoAP*) [10]. This protocol employs the basic features of HTTP to constrained networks while maintaining a low overhead. HTTP is based on the *Representational State Transfer* (*REST*) style [27]; in which the web resources are identified by URIs. Thus, CoAP enables interoperability in machine to machine (M2M) communications at the application layer through RESTful web services. Unlike HTTP, CoAP operates over the User Diagram Protocol (UDP) and applies an efficient retransmission mechanism instead of complicated congestion control as used in standard Transmission Control Protocol (TCP).

The CoAP can easily be translated to HTTP to make the seamless integration of constrained networks with the Web. To do so, CoAP proxies are employed to convert CoAP messages to HTTP packets. The main interest in making CoAP nodes part of the Internet is to allow various nodes to interact with each other using the existing web technologies.

Currently, there are several active Sensor Observation Services (e.g., GeoCENS, 52North, MapServer, Deegree, NOAA, OPeNDAP, SemSOS, *etc.*) on the Internet which follow the OGC SOS protocol. Since they are well-known data services for sensors and IoT devices, we believe the integration of CoAP and OGC SOS can benefit a great portion of IoT devices. In this section, we combined this standard protocol with CoAP in order to make CoAP nodes interoperable with other IoT components. Based on our discussion in Section 3, SOS is not originally designed for resource-constrained IoT devices. On the other hand, CoAP itself cannot validate the SOS requests because they are larger than the CoAP upper bound for request message size (1280 byes for IPv6 datagram) [10]. Therefore, one possible solution is to combine SOS and CoAP on the CoAP proxy which provides enough computational resources.

**Figure 6:**
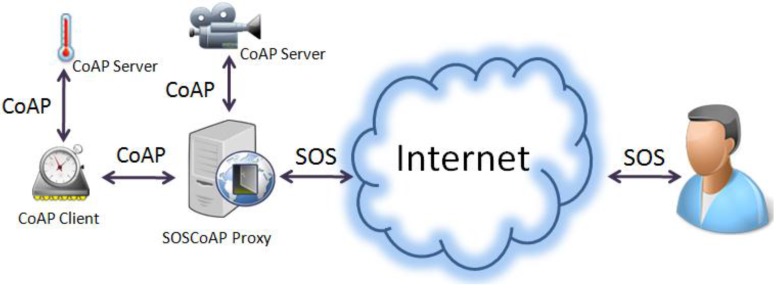
High level view of the SOS over CoAP strategy.

The major contribution of this section is that we are the first to bind the OGC SOS to the CoAP proxy denoted as *SOSCoAP proxy*. According to Figure 6, SOSCoAP proxy can communicate through CoAP regulations to the CoAP nodes (*i.e.*, IoT devices) from one side, and it can speak through the SOS standard from another side. As a result, we achieve interoperability while maintaining minimal resource consumption on IoT devices.

### 4.2. Related Work

CoAP has been already implemented in the most popular operating systems for WSNs such as, LibCoap for TinyOS [28] and CoapBlip for Contiki [29]. These research efforts mainly addressed the possibility of CoAP on target platforms with only tens of KB RAM and ROM.

There are also a few efforts to make CoAP compliant to the World Wide Web standards. For example, the *Simple Object Access Protocol* (*SOAP*) standard [30] for the data exchange of web services was bound to CoAP by Moritz *et al.* [31]. This research successfully transported SOAP messages in resource-constrained environments resulting in the deployment of web services in WSNs. However, there is a negative result in combining SOAP and CoAP as SOAP messages are encapsulated in the XML format which leads to complex message processing. Since the overhead of data transfer between SOAP-based web services is significantly higher than the RESTful web services [32,33], Castellani *et al.* [34] focused on the combination of RESTful CoAP and XML to make it more standardized. They proposed using CoAP to supply RESTful communications among applications, and the EXI (Efficient XML Interchange) format [35] to make their system more standardized according to the World Wide Web Consortium (W3C) [36]. The weakness of this design is that the interoperability issue between IoT devices was not touched on.

According to the above literature, we are not the first to argue the benefits of the CoAP and its implementation challenges, but we are the first to demonstrate the integration of this protocol to other standards of the WSNs (e.g., OGC SOS) as an interoperable infrastructure for the IoT.

### 4.3. Architecture

In this section, the proposed architecture for a CoAP-enabled IoT device is described. We then present the architecture of the SOSCoAP proxy.

#### 4.3.1. Device Architecture

**Figure 7:**
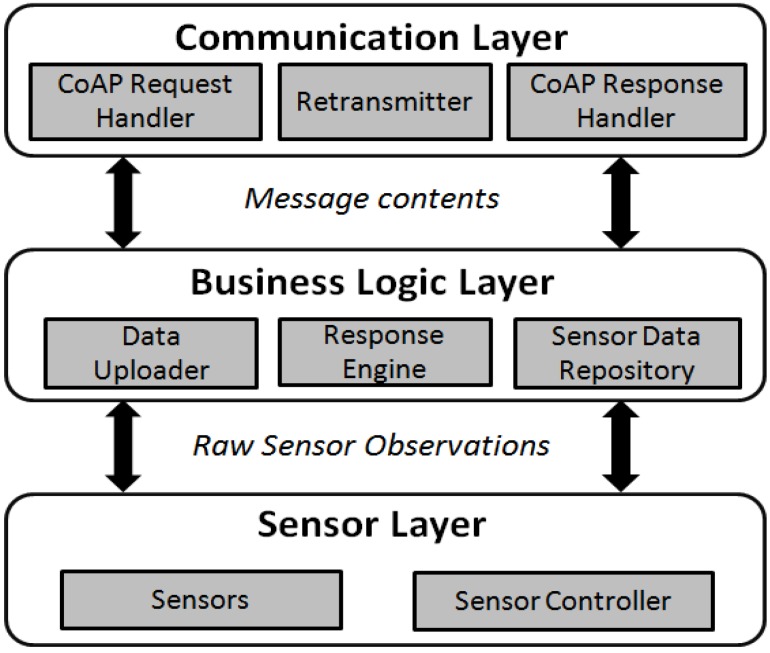
The device architecture supporting the CoAP protocol.

As depicted in Figure 7, we considered a full protocol stack for an IoT device in order to communicate through the CoAP. Likewise, the architecture consists of three layers: *Communication Layer*, *Business Login Layer*, and the *Sensor Layer*.

The sensor layer remained unchanged compared to TinySOS and PUCK. The business logic layer is partially similar to the service layer of the two prior protocols. The significant modification in this layer is the *Data Uploader* component (*i.e.*, client) in order to frequently upload the sensor observations to a pre-defined CoAP proxy. When a CoAP request is received in the communication layer, it is directly forwarded to the response engine. The response engine composes the content, and posts the message to the communication layer to be packaged in the CoAP message format. Furthermore, as a user may request historical observations, the sensor readings are stored in a sensor data repository.

More importantly, CoAP focuses on efficiency in data transmission, so the communication layer on the device is completely different from the two previous protocols. The most fundamental change is the use of UDP instead of TCP in the transport layer with a retransmission mechanism.

#### 4.3.2. SOS Integration to CoAP

**Figure 8:**
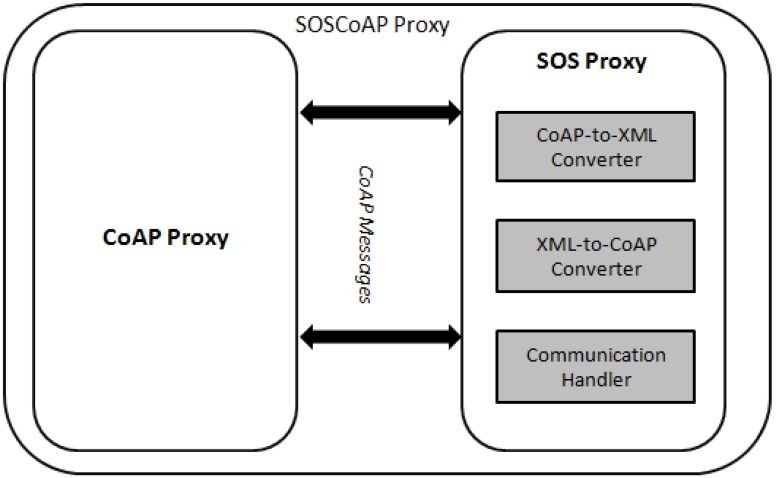
The architecture of the SOSCoAP Proxy.

SOSCoAP proxy is a regular web service within the CoAP network infrastructure as illustrated in Figure 6. One of the responsibilities of this proxy is to interconnect CoAP endpoints to users via the OGC SOS protocol. As a result, this proxy should be capable of converting the two protocols together (*i.e.*, CoAP-to-SOS, or SOS-to-CoAP). Figure 8 shows the architecture considered for the SOSCoAP proxy.

The SOSCoAP proxy consists of a *CoAP proxy* and a *SOS proxy*. For the CoAP proxy, we use jCoAP, which is an open source Java library. While the CoAP proxy is important, we do not address its components in this section as they are completely unchanged from the CoAP specification. Instead, we developed the SOS proxy that consists of three components:

- *XML-to-CoAP Converter*: This component receives the core SOS requests (GetCapabilities, DescribeSensor, and GetObservation) from the user. As those requests are encoded in heavy-weight XML, they need to be reformatted to simpler format requests encapsulated in UDP messages. The message consists of several fields defined by the CoAP specification. The body of the message has the same content of the sensor protocol already elaborated in Section 2.3.1.
- *CoAP-to-XML Converter*: This component receives CoAP messages and coverts them to SOS responses. As the CoAP messages are flat texts, they need to be encoded in XML format before being sent back to the user.
- *Communication Handler*: The communication handler checks user requests in relation to compatibility to the core SOS operations. If the request is validated, the relevant SOS response is forwarded to the user.

## 5. OGC SensorThings API

### 5.1. Introduction

In the previous sections, we clearly demonstrated that the existing protocols of Sensor Web and WSNs can be implemented on resource-constrained IoT devices. Whilst these efforts are moving the Internet of Things toward greater interoperability, they do not fit well with the processing load of IoT devices or their interconnection with other Internet nodes. In an attempt to address both deficiencies of the previous protocols, there is an ongoing effort to define a standard Web Application Programming Interface (API) for the IoT.

This API, namely *OGC SensorThings*, is an OGC candidate standard for monitoring and controlling IoT devices (sensors and actuators) over the Web. The API is built on HTTP protocols, and applies the widely-used Representational State Transfer (REST) architectural style [27] to access a system’s components. Web services complying with the REST principles are called RESTful. Since the SensorThings API is slightly different from the REST principles in terms of URI definition, we call it “REST-like”.

This API interconnects IoT services and applications over the Web through *Java Script Object Notation* (*JSON*) data format. The JSON is one of the text formats designed for representing simple data structures, data collections, and data exchange over a network connection. As an alternative to the heavy Extensible Markup Language (XML) format, we used the simple JSON format to more efficiently present the data on the server.

As a result, the SensorThings service interface differs from the existing OGC web services in terms of the REST-like interface and JSON data encoding. However, the service interface of SensorThings also leverages on the existing and widely-implemented OGC standards. For example, the capabilities part of the API service interface adapts several elements from the GetCapabilities response defined in *the OGC Web Service (OWS) Common Standard* [37] by converting the XML encoding into the JSON encoding. The OGC O&M is also reflected in the result type of the sensor observations collected on the SensorThings data service.

### 5.2. Related Work

Linking the Web and physical objects is not a new idea. With advances in computing technology, most devices are enabled with tiny web services [17,38,39]. However, the interoperability problem still exists in most of them due to the lack of a specific standard in the IoT for communication protocol and data representation.

Several systems for the integration of sensor systems with the Internet have been proposed such as SenseBox [24] and Xively, which offer a platform for people to share their sensory readings using web services. This sharing is performed by transmitting the data onto an online repository. Unlike the OGC SensorThings, these approaches exclusively support a sensing profile, and devices are considered as passive actors that are only able to push data.

Kindberg *et al.* [40] developed Cooltown project which associates web pages and URIs to people, places and things. Kindberg *et al.* also implemented scenarios in which this information could be physically discovered by scanning infrared tags in the environment. We would like to go a step further in truly making IoT devices part of the Web so that they proactively serve their functionality in an interoperable manner.

Like our REST-like web interface, T. Luckenbach *et al.* [41] and W. Drytkiewicz *et al.* [42] consider the use of REST-like architectures for sensor networks. However, to make the API interoperable, we extended the model with the use of other standards (e.g., OGC SOS, OGC SPS and OData).

In essence, the OGC SensorThings provides a REST-like web interface allowing users and application developers to apply a common API to retrieve the Things’ profiles, and sensor observations. This protocol will facilitate a generic adapter for the integration of devices to the IoT server, so that interoperability between things will become simpler.

### 5.3. Architecture

In this section, we first elaborate on the API components and its ecosystem before describing the system architecture like in the previous sections.

#### 5.3.1. API Components and Ecosystem

**Figure 9:**
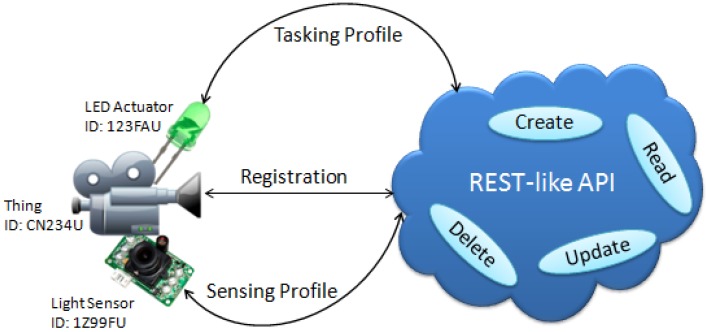
Ecosystem of the OGC SensorThings API.

The SensorThings API follows a REST-like web service interface to access the registered resources on the server. Each resource is assigned a unique identification (UID) by the server. The API supports the four basic operations of persistent storage: CREATE, READ, UPDATE, and DELETE (CRUD). The API also consists of two major profiles: *Sensing Profile* and *Tasking Profile*, which are built based on the OGC Sensor Web Enablement standards. The Sensing Profile defines an interoperable framework to manage and access sensors and observations, whilst the Tasking Profile introduces an interoperable way to submit tasks to control sensors and actuators. Figure 9 depicts the ecosystem of this API.

#### 5.3.2. System Architecture

**Figure 10:**
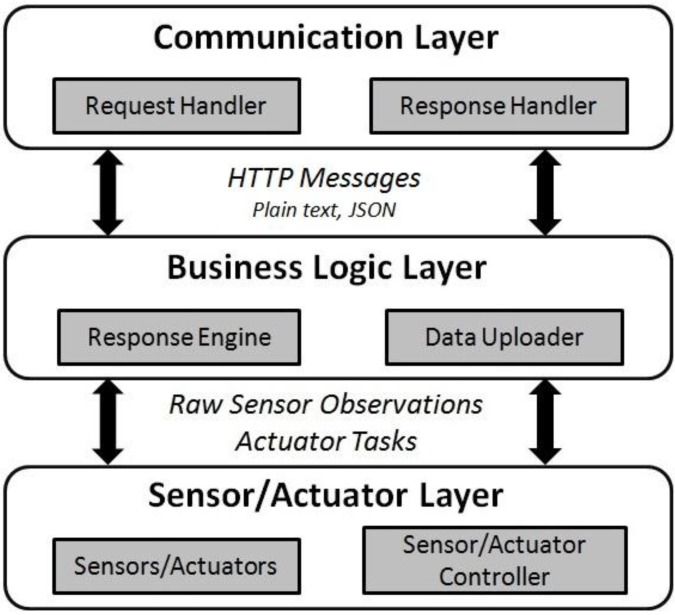
The device architecture supporting the OGC SensorThings API.

As describe in the former sections, devices supporting the OGC SensorThings API follow a system architecture to process requests and responses. In this section, we describe the proposed system architecture for IoT devices displayed in Figure 10. In this architecture, the three common layers are introduced which includes *Communication Layer*, *Business Logic Layer*, and *Sensor/Actuator Layer*.

- *Communication Layer:* Similarly with the previous protocols, the communication layer contributes to device interactions over the network. Unlike the TinySOS that uses the heavy-weight XML format, and CoAP that transmits messages in UDP packets, the OGC SensorThings API applies a basic flat format (delimited by space) in all communications except for the registration requests. When a Thing is registering itself on the server, the requests are formatted in JSON which are already hardcoded in the device’s memory. In other cases, communications are based on the flat text format that is more suitable for IoT devices to process the messages with no need for a parser.
- *Business Logic Layer:* The business logic layer simultaneously has the functionalities of both the client and the server. The client role is because a Thing demands to interact with the IoT data service in order to register itself, and to upload the sensor observations. The *data uploader* component plays the client role during device registration. This unit also sends periodic requests for publishing sensor measurements. In order to accept tasking requests from clients, the Thing should also contain a server, which is named the *response engine* component in this architecture. Similarly with the TinySOS and CoAP, the response engine reads HTTP requests buffer by buffer. After processing the requests, the task might be sent to the sensor/actuator layer, and the relevant response passed to the communication layer. Since in OGC SensorThings API, a Thing is always connected to a data service, the Thing does not need to record the sensor readings on its own memory. Therefore, unlike the other protocols, in the device architecture of this API (Figure 10), the “sensor data repository” component was completely removed from the device architecture.
- *Sensor/Actuator Layer:* The sensor/actuator layer consists of physical sensors, actuators, and their controllers. The sensor controller manages sensors and actuators. For example, the sensor controller can command a sensor to collect measurements, or give an actuator the task of carrying out an action.

## 6. Evaluation and Results

### 6.1. Introduction

The objectives of this section are as follows: (1) to benchmark the efficiency of the implemented protocols on a class-1 IoT device; (2) to provide a quantitative guideline for developers to choose the interoperable protocol that is suited to their applications. In general, this section evaluates the four standard protocols developed in this research. We assess the *performance* of those protocols on a class-1 IoT device. By performance, we mean the measurement of the degree of which a system accomplishes its functions within given constraints such as CPU speed, memory, bandwidth, and so forth [43].

In our test environment, we choose the first generation of Netduino Plus, a class-1 IoT device, as our development platform. The reason for focusing on the constrained nodes for this research is because they are more cost-effective and will be more widely deployed in the real world. By using the resource-constrained and cost-effective nodes, we can explore the lowest boundaries of the resources that are required for IoT applications. In this way, we ensure that our design choices can deliver an efficient implementation suitable for a broader application domain.

### 6.2. Performance Evaluation

This section evaluates each protocol using a service prototype (*i.e.*, server), a gateway (where applicable), and a client. The metrics selected for this evaluation are as follows: (1) code storage (EEPROM) occupation; (2) main memory (RAM) usage; (3) request length of an operation; (4) response size of an operation; and (5) response latency. In all cases except SensorThings, the tests are carried out using a Netduino Plus as the server and a PC as the client. According to Section 5, the SensorThings data service handles users’ requests and the Netduino Plus mainly acts a client which uploads its readings to the data service. In terms of network technology, both client and server are connected via Ethernet cable to the Internet in all experiments.

#### 6.2.1. Memory Occupation

The first experiment is on memory occupation (*i.e.*, ROM and RAM usage). The results obtained in this experiment demonstrate the importance of memory management in terms of resource consumption. We also included a HTTP web server in our tests as a reference. The HTTP web server is implemented in Netduino Plus and responds in a flat text format delimited by space. This web service can be a reference because it is solely developed using C# HTTP libraries with no enhancement on code efficiency.

Firstly, we measured the occupied code space after code deployment from the development environment (e.g., a PC) to the EEPROM of the Netduino Plus. The occupation of ROM can serve as an indicator of the required code’s complexity for each implementation. For example, according to Table 1, the OGC SensorThings API and SOS over CoAP need more ROM in comparison to the other implementations, because both not only need to handle server-side operations but also need to support client-side functions. The simple web service is third in terms of ROM usage as the classes and libraries in the C# .Net Micro Framework consume a considerable amount of code storage [44]. Compared to the simple web server, TinySOS is more efficient for two reasons: (1) instead of using the C# .Net Micro Framework’s libraries, we implemented our own HTTP libraries; (2) we recorded the XML responses on the micro SD card instead of ROM. The OGC PUCK is the most efficient protocol in terms of ROM usage because PUCK specification does not require any heavy parser (e.g., XML parser, JSON parser), retransmission mechanism (e.g., CoAP-To-HTTP), and data uploader component. Although the OGC PUCK requires PUCK memory, SensorML and driver code, we are able to use the device’s permanent memory (micro SD card) to keep the necessary data.

Moreover, Table 1 shows the amount of RAM allocated at runtime for each implementation. A code with a small memory footprint would allow adding extra capabilities, such as resources that the server could provide to clients. Although PUCK occupies the least code space, this protocol is highly inefficient in RAM usage. It is possible that the memory management unit or data transceiver of the Bluetooth module requires more memory in comparison to other components of this protocol. After the OGC PUCK, the TinySOS consumes a lot of RAM possibly due to the XML parser and request validator units. SOS over CoAP and OGC SensorThings are similar in terms of RAM usage. The simple web server performs better than others in this experiment since it is simple in relation to request validation and response generation.

**Table 1:** RAM and ROM memory occupation.

	Simple Web Service	PUCK over Bluetooth	TinySOS	SOS over CoAP	OGC SensorThings
**ROM (KB)**	16.08	8.48	11.72	29.13	26.11
**RAM (KB)**	9.54	13.15	11.33	10.36	10.21

#### 6.2.2. Request Size

**Figure 11:**
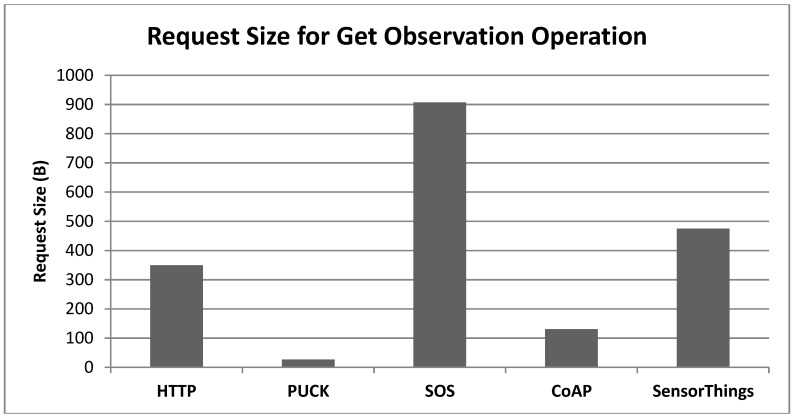
Request size evaluation for the get observation request.

Both IoT devices and the network they use are highly constrained [2]. This means that the payload packet size is very important. To identify the efficiency of the above standard protocols, we recorded the request size generated for a specific use case (*i.e.*, get one sensor measurement) that is possibly the most widely used. To do this, we use Wireshark, a network protocol analyzer software for all tests except PUCK. That is Wireshark is unable to monitor the serial ports that are the communication ports of the PUCK. Thus, to measure the PUCK request size, we simply count the characters of its plain text request. According to Figure 11, PUCK generates the smallest request since the request is made of a short string of characters with no header, description or complicated format. In addition, CoAP request is at least 67% smaller in comparison with other Internet-based protocols. This efficiency is due to using UDP instead of TCP in the transport layer, which makes the header size much smaller. The simple web service communicates through HTTP GET request with no request content. Therefore, only the header features of the HTTP GET request (350 bytes) are calculated for the simple web service. The OGC SensorThings requires several parameters embedded in the request body apart from the header features. Therefore, SensorThings is ranked after the HTTP protocol in this experiment. In contrast, the requests of the SOS protocol are at least 47% larger than other protocols since they are packaged in XML format. In order to ensure that the tested SOS request is compatible with the OGC SOS standard, we used a test client tool developed by 52 North SOS.

#### 6.2.3. Response Length

**Figure 12:**
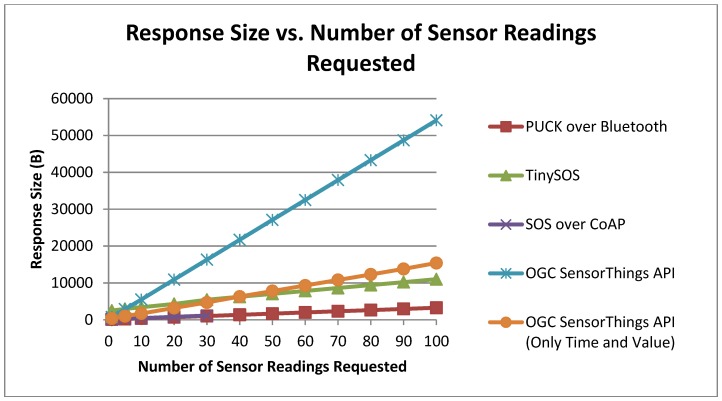
Response size *vs.* the number of sensor readings.

Apart from the requests comparison among the standard protocols, we also evaluated the response length generated by our implementations. Figure 12 depicts the response length trend of different implementations *versus* the number of sensor readings requested (from 1 to 100). Since the specification of OGC SensorThings conveys the sensor related requests to a REST-like data service, we send the get observation request to that data service (a regular PC) instead of Netduino Plus. According to Figure 12, OGC SensorThings and TinySOS provide larger responses in comparison to other protocols. One explanation for this difference can be the output formatting which is in JSON and XML, respectively. After looking at the responses generated by OGC SensorThings data service, we identified several JSON attribute-value pairs (e.g., observation ID, request type, feature of interest, sensor profile, and data stream information) that were repeated in all sensor readings. Based on the capabilities of the SensorThings data service, we were able to retrieve only the sensor measurement and the observation time in JSON format. As a result, the response length was calculated to be 71% less in average compared to the previous responses of the SensorThings API. In contrast, TinySOS follows the OGC SOS specification for response generation by embedding the observation values and times in the existing response file. Accordingly, the response size will not be as large as the OGC SensorThings protocol with repetitive attribute-value pairs. As a result, end users can simply parse the SensorThings responses by a JSON parser whilst for the TinySOS responses, a new parser needs to be developed in order to extract the required data from the XML file.

**Figure 13:**
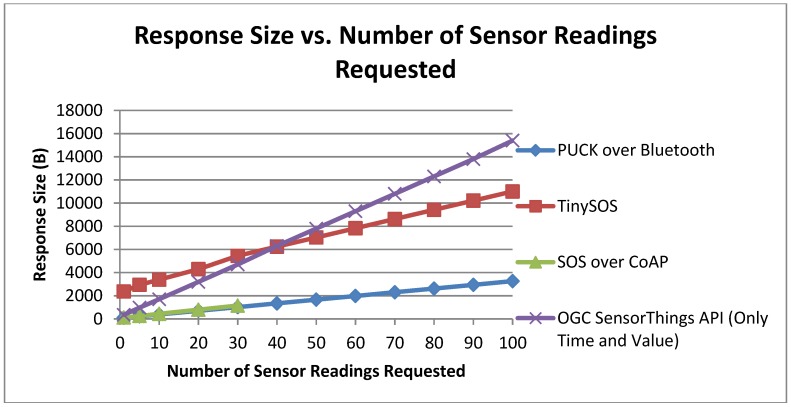
Response size *vs.* the number of sensor readings (removed the OGC SensorThings trend).

To better understand the trends of other implementations, we removed the OGC SensorThings trend in Figure 13. The SOS over CoAP and PUCK over Bluetooth follow each other closely since the protocols defined to retrieve sensor readings are the same for both. According to the CoAP specification [10], CoAP messages should not exceed 1024 bytes. That explains why the green line representing SOS over CoAP in Figure 13 has not gone further than point 30 for which the response size was 1019 bytes. However, the required response header of CoAP makes the CoAP response size a bit larger than the output from PUCK for cases with equal number of sensor readings.

According to Figure 13, TinySOS and OGC SensorThings API generated the same response size for forty senor observations. Due to the fact that the observation values and time are the same for the two protocols, we can conclude that the size of the XML tags of the SOS response is equal to the total length of the JSON attribute-value pairs of the SensorThings response (*i.e.*, “time”, “result value”, “self-link”).

#### 6.2.4. Response Latency

**Figure 14:**
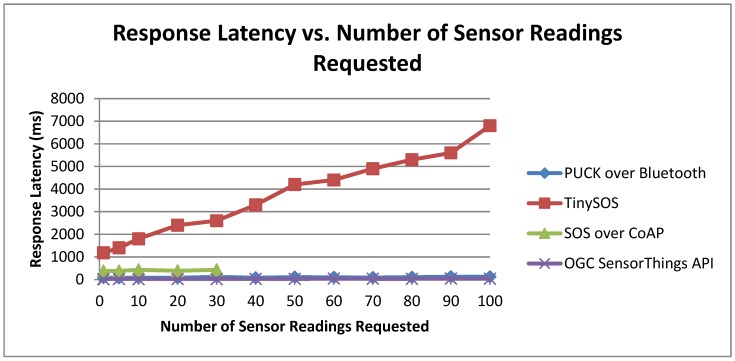
Response latency *vs.* the number of sensor readings.

To conclude our performance evaluation, we recorded the end-to-end response latency. The experiment was conducted by a PC client to retrieve sensor data from a Netduino Plus-based service or from a PC-based IoT data service. We define latency as the time elapsed from the moment the PC client sends a request to the moment it receives the response. Figure 14 shows the latency trend based on our experiments. Each point on Figure 14 represents the latency value of successful request/response transactions. The number of sensor readings ranges from 1 to 100. In this way, the differences between the other implementations can be better appreciated. Low latency values can notably improve the user experience and benefit the implementations that work in real-time.

TinySOS behaved worse than others in this experiment as its communications are in XML data encoding. Thus, the Netduino Plus server has to parse the XML request, read the XML response file from the micro SD card, embed sensor reading(s) into the response body, and forward the XML file to the client. All these functions are performed on a device with 48 MHz CPU speed and 28 KB memory which lead to high latency.

**Figure 15:**
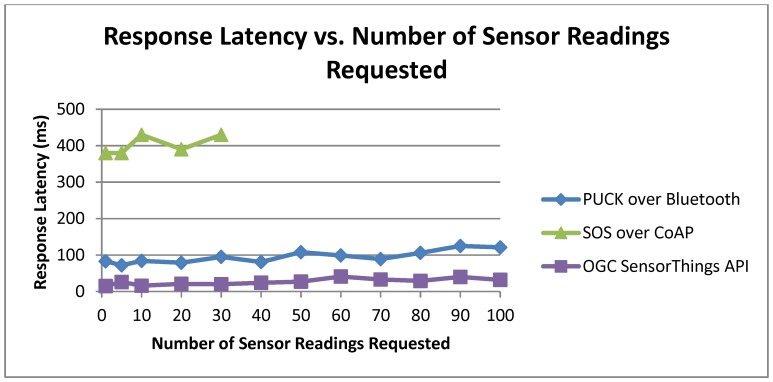
Response latency *vs.* the number of sensor readings (removed TinySOS).

Figure 15 removed the TinySOS trend in order to determine the behavior of other implementations. The SOS over CoAP has more latency than PUCK since the CoAP communicates over the World Wide Web. As we explained in Section 6.2.3, CoAP stopped at point 30 because of the CoAP limitation for the message size. Due to the fact that the data service of the SensorThings is a regular PC, if we ignore this protocol, the PUCK over Bluetooth is the most efficient implementation in this experiment. For the PUCK evaluation, we applied Device Monitoring Studio software in order to monitor the serial port of the PC. Since PUCK over Bluetooth is a wireless protocol, the distance between the pairs affects the response latency. In our experiments, the Netduino Plus (server) and the notebook (client) were placed close to each other (less than 1 m).

## 7. Conclusions and Future Work

### 7.1. Conclusions

We conclude this paper in the final section by summarizing the research that has been carried out and outlining the conclusions drawn from the result. We also comment on the limitations and propose areas for future work.

We began by choosing a class-1 IoT object as categorized in the framework of Bormann *et al.* [2] for our development platform. Firstly, we equipped the class-1 IoT device with a Bluetooth transceiver in order to establish wireless network within a limited range. We standardized its connection by means of OGC PUCK. Due to the fact that Internet access is a key requirement for IoT devices, we applied additional software components to enhance this functionality for the Bluetooth-enabled PUCK instrument.

In the second stage, we removed the intermediary gateway in the path between user and IoT device by developing a web service on a Thing itself. Since device owners or manufacturers might have their own design for data representation, we introduced a lightweight version of the OGC SOS, TinySOS. As a result, the sensor measurements could be accessed remotely in a standardized way simply through a web browser.

In accordance to the complicated nature of the OGC SOS, we proposed another approach which was more suitable for the class-1 IoT devices. As such, the third contribution of this research was integrating CoAP into the OGC SOS for which the SOS operations were processed on the CoAP proxy with enough computational resources.

Due to the UDP transmission, CoAP could not establish a direct connection to the Internet components without the deployment of CoAP proxies. As the IoT will eventually follow the Internet protocol suite model, it is recommended to adjust network connections to be compatible with the Internet standard protocols. As a result, we designed a new application programming interface called OGC SensorThings API. The use cases of this API started with device registrations to the service. For the sensing devices, registration information contained the phenomenon that was observed. After registration, sensing devices could start uploading their observations to the data service. From the point of view of tasking, actuators could also register and publish their tasking capabilities to the data service. As a result, users were able to access those observations and also send controlling tasks to the devices through the service. All the communications with the data service followed the REST-like architecture.

Finally, the four implementations on a class-1 IoT object (Netduino Plus) were assessed comparatively. Each implementation was evaluated according to memory occupation (RAM and ROM), request size, response length and response latency. As a case study, we embedded multiple meteorological sensors, sound pressure sensor and LED actuator to our Netduino Plus in order to demonstrate how different components work together.

In summary, each of the protocols discussed in this research has its own negative and positive attributes which can effectively influence the protocol selection for application developers. According to the evaluation results, TinySOS and PUCK appeared significantly efficient in terms of ROM usage whilst the SOS over CoAP and the SensorThings API performed slightly better for RAM usage. Thus, PUCK and TinySOS are better candidates for memory-constrained applications. In terms of bandwidth efficiency, the experiments nominated CoAP and SensorThings which had small request and response sizes. Lastly, for real-time applications, TinySOS is not recommended at all since handling large XML documents is time-consuming for resource-constraint devices.

### 7.2. Future Work

This paper takes a practical approach to the interoperability in the Internet of Things. There are several ways in which this research can be improved and extended. In this section, some of the major issues that can later be investigated and guidelines of the future work are addressed.

First of all, the aforementioned interoperable protocols follow the client-server architectural style which has the single point of failure (SPOF) issue [45]. In order to address this issue, one potential solution is to design a peer-to-peer (P2P) architecture as it has been proven reliable and effective. In this case, devices can form an overlay network to discover resources and forward requests; so a centralized component such as the sensor registry service, CoAP proxy and SensorThings data service would be no longer needed.

For this research, Bluetooth and Ethernet were considered as the network enablement technologies for IoT devices. Since Wi-Fi is dominant in network communications [46], the study on Wi-Fi communications in the IoT is strongly recommended. One immediate issue in Wi-Fi connection is the transmission of network configuration to the IoT device which has no display equipment and input peripheral.

In addition to Wi-Fi as wireless enablement for the IoT, research should be started on improving energy saving on the IoT devices. The first assumption for this research was IoT devices having unlimited power resources; however this assumption may not be true in many cases. Some sensor nodes will be battery-operated [26], so energy is perhaps the most notable constraint for the IoT devices. Furthermore, achievement in Wi-Fi connection of IoT objects leads to removing wires and cords from devices. Therefore, their battery charge must be efficiently conserved to extend the life of the individual sensor nodes, and consequently the entire IoT network.

One of the missing metrics in Section 6 was the evaluation of CPU usage in different protocols. The CPU usage refers to how much work a device’s processor is doing. The main reason that we did not focus on this criteria in our evaluations was because there was no complex computation performed on the device. For example, in none of the four protocols, the device did not implement any JSON parser or XML parser which is heavy-weight for class-1 IoT devices. However, it is worth to compare the CPU usage for the existing workload of those protocols in the future.

More potential future work pertains to privacy and security for IoT devices. We efficiently implemented existing security and privacy mechanisms of the information technology and computer networks on class-1 IoT devices [47]. Although an acceptable level of secure connection in IoT can be achieved, we believe IoT would require specific rules and mechanisms for the successful implementation of this approach.

## Figures and Tables

**Figure A1:**
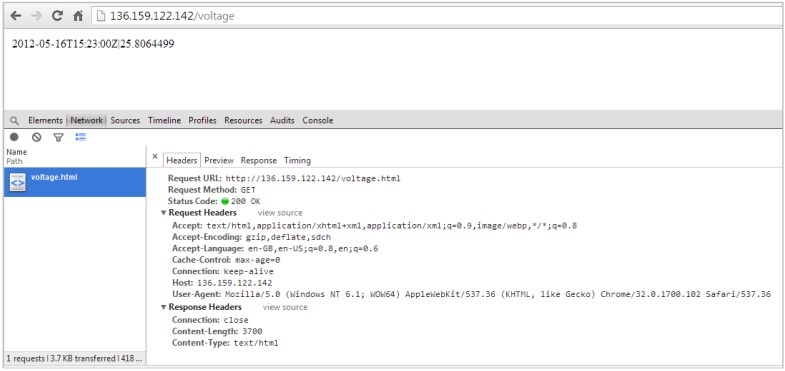
HTTP GET request and response to the simple web server through the Internet.

**Figure A2:**
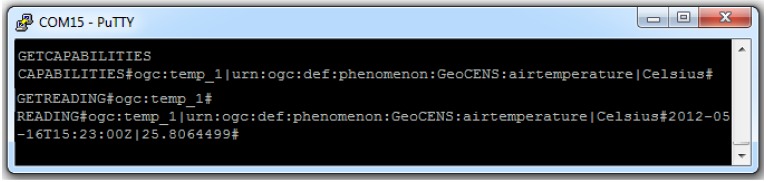
GETREADING request and response to the PUCK-enabled Netduino Plus through Bluetooth.

**Figure A3:**
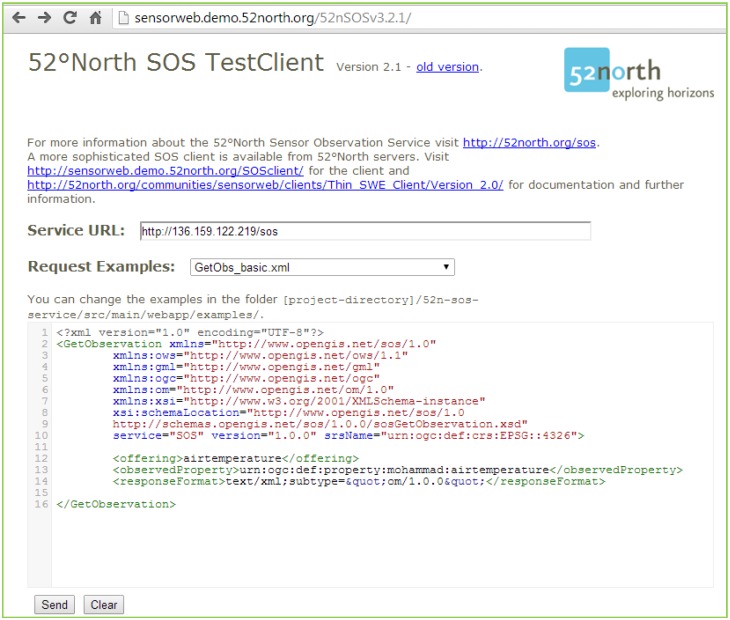
GetObservation request to TinySOS using the 52 North test client tool.

**Figure A4:**
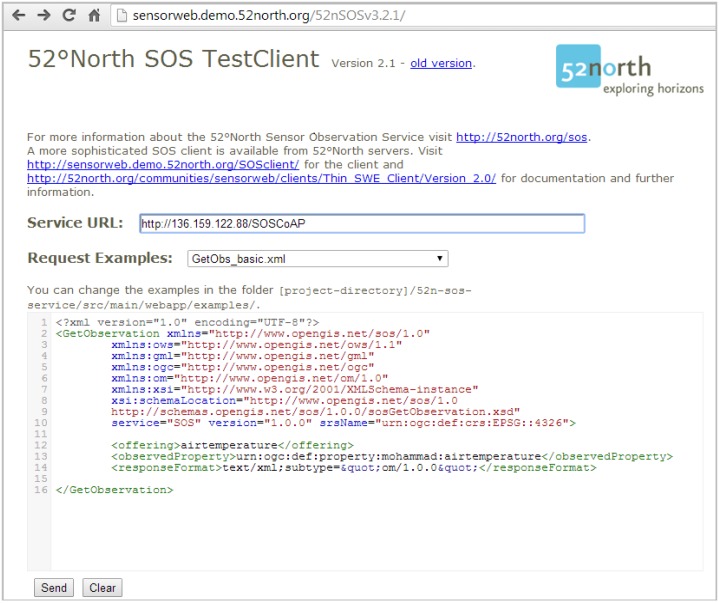
GetObservation request to the SOSCoAP proxy using 52 North test client tool.

**Figure A5:**
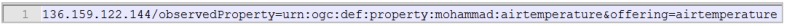
GetObservation request to a CoAP server (*i.e.*, Netduino Plus) using the SOSCoAP Proxy.

**Figure A6:**
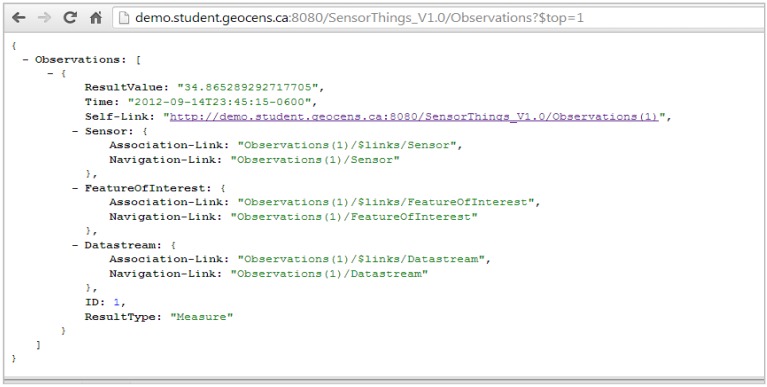
HTTP GET request and response to the OGC SensorThings through the Internet.

**Figure A7:**
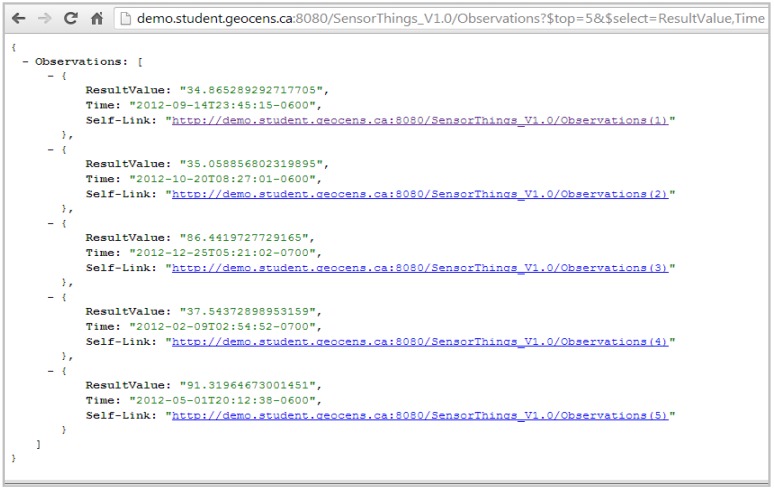
Summarized response of the SensorThings.

**Figure A8:**
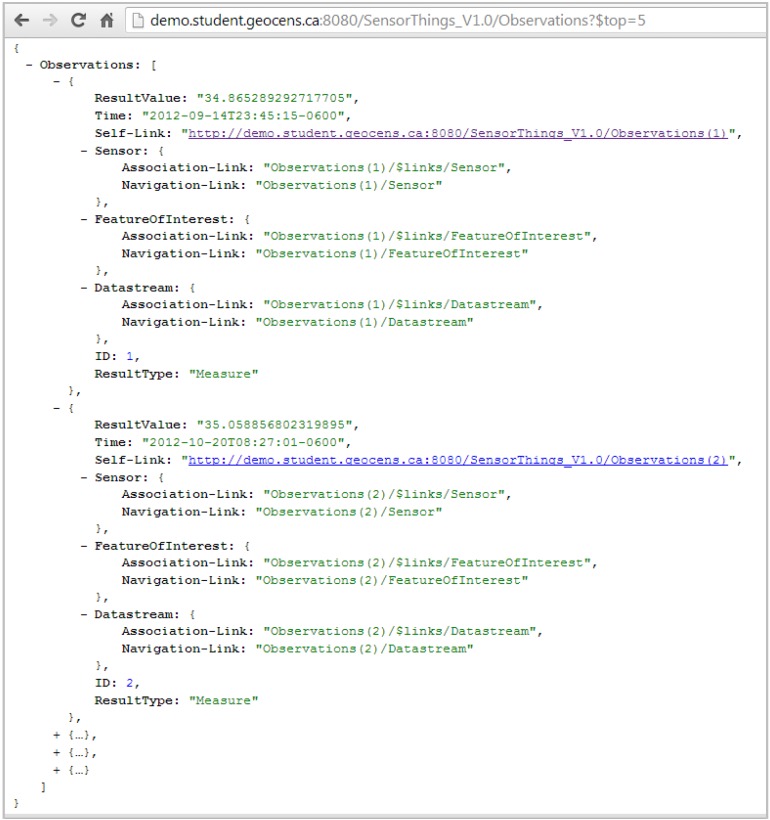
Response of the SensorThings to multiple readings request.

## Appendix A

### A.1. Sample Requests and Responses Used in Section 6.2

In this section, requests and responses of the get observation operation for each of the four implemented protocols are presented.
